# Macrophyte communities as indicators of the ecological status of drainage canals and regulated rivers (Eastern Poland)

**DOI:** 10.1007/s10661-022-09777-0

**Published:** 2022-02-22

**Authors:** Monika Tarkowska-Kukuryk, Antoni Grzywna

**Affiliations:** 1 Department of Hydrobiology, University of Life Sciences in Lublin, B. Dobrzańskiego 37, 20-262 Lublin, Poland; 2 Department of Environmental Engineering and Geodesy, University of Live Sciences in Lublin, Leszczyńskiego 7, 20- 069 Lublin, Poland

**Keywords:** Macrophyte Index for River, Hydromorphological Index for River, Chemical parameters, Artificial watercourses

## Abstract

Macrophytes are one of the biological elements for assessing the ecological status. Macrophyte communities were studied in six artificial (drainage canal and ditches) or modified watercourses (regulated rivers). In order to assess the ecological status of the watercourses, studies were carried out with the use of the Macrophyte Index for Rivers (MIR) proposed in the Water Framework Directive. Macrophyte communities were mainly represented by helophytes (12 species in total), and the highest percentage area cover (50–75% of the site) was observed for pleustophytes (*Lemna spp.*). Macrophytes communities were affected by the gradients of hydromorphological (canal modification, flow type, and shading) and parameters water quality (nutrients and water transparency). The relationships were highly species-specific. In accordance with the MIR values, the ecosystems were classified as having poor (drainage canal), moderate (regulated rivers), or good (drainage ditches) ecological status.

## Introduction

Macrophytes are one of the biological elements for assessing the ecological status of rivers in compliance with the EU Water Framework Directive (Directive 2000/60/EC). As major primary producers, they play a crucial role in the trophic structure of river ecosystems, interacting with higher trophic levels by providing food and refuge for macroinvertebrates and fish (Elosegi et al., 2018; Ferreiro et al., 2011; Huggins et al., 2004). Macrophytes also affect water quality, through their involvement in nutrient cycling and sediment resuspension (Abrahams, 2008; Baattrup-Pedersen & Riis, 1999; Kleeberg et al., 2010).

In Europe, several indices based on macrophytes have been developed to assess water quality (mainly trophic status), e.g., the British Mean Trophic Rank (MTR) (Holmes et al., 1998), the German Trophic Index of Macrophytes (TIM) (Schneider & Melzer, 2003), and the French Biological Macrophytes Index for Rivers (IBMR) (Haury et al., 2006). In Poland, the use of biological elements is defined by a Regulation of the Ministry of the Environment (Journal of Laws, 2019), according to which the Macrophyte Index for River (MIR) (Staniszewski et al., 2006; Gebler et el., 2018; Szoszkiewicz et al., 2020) should be used to assess water quality in rivers. MIR reflects river degradation, especially eutrophication. This method is consistent with most European methods, such as MTR, IBMR, and EU-STAR project methodology (Erba et al., 2006).

Macrophytes are widely used to assess the environmental conditions of different types of river ecosystems (Hroudová & Zákravsky, 1999; Dodkins et al., 2005; Demars & Edwards, 2009; Kopeć et al., 2010; Kuhar et al., 2010; Ceschin et al., 2015, 2020; Papastergiadou et al., 2014). However, studies conducted so the role of macrophyte communities as indicators of the ecological status of artificial (drainage ditches and canals) and heavily modified (rivers with rectified beds) watercourses are scarce and insufficient.

Infrastructure development in river valleys causes severe modification of river channel form and processes. The immediate hydrological alterations caused by dams and flow regulation alter the form of river channels and thus the composition and extent of aquatic vegetation (Bejarano et al., 2018; Jansson et al., 2000; Jones et al., 2020; Merritt & Wohl, 2002; Ramos-Merchante et al., 2021; Riis & Sand-Jensen, 2001; Tombolini et al., 2014). Regulated rivers usually have significantly lower richness (Nilsson et al., 1997) and different floristic composition, with a higher number of sporadic species and annuals (Bejarano et al., 2020; Netten et al., 2011). Artificial watercourses such as drainage ditches and canals are typical for many countries (e.g., Germany, the Netherlands and England). These artificial aquatic systems have an intermediate status between flowing and stagnant water bodies, with shallow, relatively clear water, a soft muddy bottom and very low flow rates. These environmental conditions favor the development of aquatic vegetation; therefore, canals are often a refuge for endangered and rare macrophyte species (Langheinrich et al., 2004; Dodkins et al., 2005; Caffrey et al., 2006; Gething et al., 2020; Papastergiadou et al., 2014; Hachoł et al., 2019).

In this article, we present the results of a case study of Western Polesie (eastern Poland) concerning the use of macrophyte communities to evaluate the trophic state of regulated rivers and drainage canals. These anthropogenic water ecosystems are typical for the landscape of the region. During the 1960s and 1980s, in order to enlarge the arable land area, the hydrological conditions in Western Polesie were significantly transformed, mainly through large-scale land reclamation, the creation of a very dense network (3 km*km^2^) of drainage ditches, and regulation of rivers (rectification of riverbeds). During the years 1955–1960 the Wieprz-Krzna Canal (WKC), the longest drainage canal in Poland (140 km), was built in the area. Part of the WKC system consisted of lakes converted into storage reservoirs and some artificial reservoirs. All drainage canals were maintained regularly until 1990, but since then have been completely neglected. The lack of maintenance activity in the last 25 years has enabled the development of abundant and diverse macrophyte communities (Chmielewski, 2009).

Our study focuses on the response of macrophytes to hydromorphological and physicochemical conditions of these artificial and heavily modified watercourses. We expected to find a relationship between macrophyte composition, environmental parameters (temperature, pH, conductivity, total suspension, dissolved oxygen, planktonic chlorophyll-*a* and nutrients), and the type of bottom substrate. The value of the paper is that it presents comprehensive monitoring results concerning all the mentioned groups of parameters on selected drainage canals and regulated rivers. Thus, coupling macrophyte data, hydromorphology, and water quality and finding their relationships might present a novel approach. Another interesting moment is that it presents monitoring of abandoned/poorly managed canals (for 25 years) and regulated rivers.

## Material and methods

### Study area 

The study was conducted in the Parczew forests (PLB060006), a Nature 2000 Special Protected Area (SPA). The area was designated according to the Regulation of the Ministry of the Environment (Journal of Laws, 2004) on special bird protection areas. Macrophytes were investigated in six watercourses: the Wieprz-Krzna Canal (start 51^o^31′34.3″ N, 23^o^04′14.5″ E; end 51^o^31′34.7″ N, 23^o^04′15″ E), the drainage ditches—Uhnin (start 51^o^34′51.4″ N, 23^o^05′00.0″ E; 51^o^34′51.8″ N, 23^o^05′00.6″ E), Sosnowica (start 51^o^31′33.8″ N, 23^o^04′41.4″ E; end 51^o^31′34.2″ N, 23^o^04′41.9″ E) and Ambona (start 51^o^33′55.1″ N, 22° 52′43.0″ E; end 51^o^33′55.7″ N, 22° 52′43.3″ E), two rivers with rectified beds—Ochoża River (start 51^o^33′48.3″ N, 22^o^52′37.6″ E; end 51^o^33′48.7″ N, 22^o^52′37.9″ E) and Piwonia River (start 51^o^31′34.7″ N, 23^o^04′25.6″ E; end 51^o^31′35″ N, 23^o^04′25.9″ E). For each of the watercourses, a representative 100-m-long reach was chosen for the macrophytes survey.

### Macrophytes survey

Macrophytes were collected during the vegetation season (June–August 2018 year) using the Macrophyte Method for Rivers (Jusik et al., 2020). At each site (watercourse), macrophytes were examined by wading along a 100-m reach. Macrophytes were surveyed every 10 m along transects connecting the banks. It was assumed that one transect was equal to one sample; thus, we obtained 10 samples per watercourse. We identified all vegetation forms (submerged, free-floating, amphibious, emergent, filamentous algae, liverworts, and mosses) and macrophytes rooted or attached to a river bank (submerged for more than 10 months of the year). The mean cover of a macrophyte species was estimated using a nine-point scale: < 0.1% (1), 0.1–1% (2), 1–2.5% (3), 2.5–5% (4), 5–10% (5), 10–25% (6), 25–50% (7), 50–75% (8) and > 75% (9). The macrophytes were classified into six categories: emergent plants (helophytes), floating-leaved rooted plants (nymphoides), floating-leaved unrooted plants (pleustophytes), submerged plants (elodeids), mosses and liverworts (bryophytes) and filamentous algae.

The Macrophyte Index for Rivers was calculated (Szoszkiewicz et al., 2020):

1 $$\mathrm{MIR }=[\sum_{i=1}^{\mathrm{N}}\mathrm{L}i*\mathrm{W}i*\mathrm{P}i/{\sum }_{i=1}^{\mathrm{N}}\mathrm{W}i*\mathrm{P}i]*10$$

where N—number of species at the sampling site, P*i* –% cover for the *i* taxon, W*i*—weighting factor for the *i* taxon. This is a measure of ecological tolerance. Its values ranged from 1 (eurytopic species) to 3 (stenotopic species). L*i*—indicator value for the *i* taxon.

MIR values were applied according to the Regulation of the Ministry of the Environment (Journal of Laws, 2019). The ecological potential of the watercourses was classified according to MIR values for artificial or heavily modified watercourses of Type 17 (small lowland peatland rivers) as follows: MIR ≥ 46.8 (very good); ≥ 36.6 (good); ≥ 26.4 (moderate); ≥ 16.1 (poor) and < 16.1 (bad).

### Water sampling

Water samples for chemical analysis were collected simultaneously with as the macrophyte survey. Ten replicate samples were taken at each site on each occasion. Water temperature (T), pH, conductivity (EC) and dissolved oxygen (DO) were measured in situ using a Multi-340i WTW microcomputer analyzer. The following were determined in the laboratory: total nitrogen (TN) and ammonium nitrogen (N-NH_4_)—PC AQUALYTIC spectrophotometer; nitrate nitrogen (N-NO_3_), total phosphorous (TP), phosphate (P-PO_4_)—LF-300 Slandi photometer; chemical oxygen demand (COD)—bichromate method; total suspension solid (TSS)—gravimetric method (Grzywna et al., 2015). Planktonic chlorophyll-*a* (Chl) as an indicator of phytoplankton biomass was determined by spectrophotometry following 24-h extraction with 90% acetone in the dark.

### Hydromorphological River Index

The macrophyte studies were supplemented with the hydromorphological assessment of each site. Hydromorphological studies of rivers were carried out on the basis of the River Habitat Survey method (Szoszkiewicz et al., 2017). It is a system for assessing the nature of the habitat and the quality of watercourses using morphological and hydrological parameters. The analyzed parameters include: longitudinal profile, hydrotechnical structures, land use of the river valley, forest cover, communication with the river valley, physical attributes of the river bed (type of flow, morphological elements of the bottom and banks, bed material), types of vegetation in the river bed. Habitats were assessed by collecting observational data over a 500-m-long river section and its corridor extending 50 m outside on each side. Observations are conducted at two different scales: i) at perpendicular transects every 50 m, ii) continuously along whole the 500-m survey site. These studies provided a lot of environmental information about the examined points. Based on the hydromorphological data, the Hydromorphological Index for River (HIR) was calculated.

### Data analysis

According to the Shapiro–Wilk normality tests, the macrophyte and environmental data follow a normal distribution; one-way ANOVA was used to verify the influence of habitat type (canal, drainage ditch, regulated river) on the species richness and cover of macrophytes and to determine the influence of the hydromorphological conditions on macrophyte species. Pearson`s correlation coefficients were calculated to determine the relationships between macrophyte species and environmental parameters. The analysis was performed using STATISTICA 10.0.

## Results and discussion

### Hydromorphological characteristic

All studied watercourses are characterized by the following identical hydromorphological parameters: longitudinal profile, elements of the bottom, land use of the valley area. Border elements (Table 1). Most often, small hydraulic structures in the form of gates are located on the analyzed watercourses. Ditches and rivers are located in peat bogs. Only in the case of DC there is a weir on it and the water course does not run through the peat bog. The greatest differentiation of the characteristics occurs in the case of bottom material, type flow, and cross-section. There are also slight differences in the case of tree plantings (Table 1).

### Macrophyte structure

In the ecosystems studied, we identified 22 macrophyte species (Table 2) representing four ecological groups: pleustophytes (4 species), nymphoides (2 species), elodeids (4 species) and helophytes (12 species). The total number of macrophyte species differed significantly (ANOVA, df = 5, *F* = 95.63, *p* < 0.001) between sites, ranging from 2 (DC) to 12 species (DD_1). The percentage cover of the macrophyte species clearly depended on the site (watercourse) (*F* = 5.11, *p* = 0.002). The highest cover, over 75% of the area of the site, was observed for the pleustophytes *Lemna minor* (DD_1, DD_2, RR_2) and *L. trisulca* (DD_2, RR_2), and for the elodeid *Stratiotes aloides* (DD_1). The lowest cover, between 1% and 2.5%, was noted for the helophyte *Rumex hydrolapathum* (DD_2) and the elodeids *Potamogeton pectinatus* (DC) (Table 2).

**Table 1:** Hydromorphological characteristic of the studied sites.

Site	DC	DD_1	DD_2	DD_3	RR_1	RR_2
Longitudinal profile	straight	straight	straight	straight	straight	straight
Hydrotechnical structures	medium	small	small	small	small	no
Land use of the valley area	grassland	grassland	grassland	grassland	grassland	grassland
Communication with the valley	no	peatland	peatland	peatland	peatland	peatland
Type of flow	fast	slow	slow	slow	fast	laminar
Elements of the bottom	no	no	no	no	no	no
Border elements	stable	stable	stable	stable	stable	stable
Bottom material	mud	silt	silt	silt	sand	sand
Cross-section	strengthened	profiled	profiled	profiled	mild	steep
Tree plantings	no	shrubs	shrubs	shrubs	no	no
HIR	0.28	0.25	0.25	0.25	0.33	0.35

DC – Wieprz-Krzna Canal, DD_1 – drainage ditch Uhnin; DD_2 – drainage ditch Sosnowica; DD_3 – drainage ditch Ambona; RR_1 – regulated River Ochoża; RR_2 – regulated River Piwonia

The observed species composition of macrophytes was typical for reservoirs of anthropogenic origin or subject to the inflow of nutrients from the catchment area. Phragmites australis is an indicator species for hypertrophic waters with periodic oxygen deficits. This species reacts very clearly to the increase in the concentration of biogenic compounds in water, increasing its biomass. *Ceratophyllum demersum* is a species that is not rooted in the bottom and is able to take up inorganic forms of phosphorus from the water, and therefore, it can compete with phytoplankton. The occurrence of *Lemna minor* is limited to water ecosystems with an increased content of biogenic elements. These species form a compact plant cover on the water surface, negatively affecting the oxygen concentration in the water (Grzywna et al., 2015).

The diversity of the macrophyte communities of the watercourses was low, at a level typical for human-impacted or heavily modified streams. Comparable species richness has been observed in European mountain streams in Slovakia (Hrivnák et al., 2010, 2012) and north-western Slovenia (Kuhar et al*.*, 2010) as well in lowland rivers and ditches of southern England (Riley et al., 2018; Williams et al., 2004). More macrophyte species have been observed in lowland streams in north-western Europe (Baatrup-Pedersen et al., 2006; Halabowski & Lewin, 2020). In general, the diversity of macrophyte communities in natural and modified watercourses is the result of the synergistic effect of environmental and anthropogenic factors. In man-made canals, external factors, such as in canal and bankside maintenance, dredging, aquatic weed control and water flow regulation, influence plant composition and distribution (Demars et al., 2014; Hachoł et al., 2019). The watercourses we studied have not been maintained for the last 25 years. No plant management or weed control has been practiced in these ecosystems. Therefore, we assumed that the macrophytes of the drainage ditches, drainage canal and regulated rivers are affected by the gradients of hydromorphological and environmental conditions.

The MIR ranged from 18.0 (DC) to 39.5 (DD_2), which allowed three different ecological statuses to be assigned to the ecosystems. The Wieprz-Krzna canal was classified as having poor ecological status, the Uhnin, Sosnowica and Ambona drainage ditches (DD_1, DD_2, DD_3) had good ecological status, and the ecosystems of the regulated rivers Ochoża and Piwonia (RR_1, RR_2) had moderate ecological status.

### Environmental variables

Abiotic conditions displayed marked variability between watercourses (Table 3). The drainage ditches, drainage canal and regulated rivers differed significantly in conductivity (ANOVA, df = 5, *F* = 24.41, *p* < 0.001), concentrations of total suspension (*F* = 16.15, *p* < 0.001), dissolved oxygen (*F* = 14.34, *p* = 0.002), N-NH_4_ (*F* = 7.78, *p* = 0.009), N-NO_3_ (*F* = 69.57, *p* < 0.001), P-PO_4_ (*F* = 11.88, *p* = 0.003) and planktonic chlorophyll-a (*F* = 15.73, *p* = 0.001).

**Table 2:** Cover of the macrophyte species

	Sites	DC	DD_1	DD_2	DD_3	RR_1	RR_2
Pleustophytes							
*Hydrocharis morsus-ranae*	3		4	4	7		
*Lemna gibba*	1		6				
*Lemna minor*	5		8	8	7	6	8
*Lemna trisulca*			4	8	7	6	8
Nymphoides							
*Nuphar lutea*	1						5
*Potamogeton natans*	1		6				
Elodeids							
*Ceratophyllum demersum*	5	4		7	7	6	7
*Elodea canadensis*	2					6	5
*Potamogeton pectinatus*	1	3					
*Stratiotes aloides*	1		8				
Helophytes							
*Acorus calamus*	1			4			
*Calla palustris*	1			5			
*Carex rostrata*	2		5	4			
*Carex vesicaria*	1		5				
*Equisetum palustre*	1			4			
*Glyceria maxima*	2		4	4			
*Juncus effusus*	1		5				
*Mentha aquatica*	1					5	
*Phragmites australis*	4		7		7	7	5
*Rumex hydrolapathum*	2			3	6		
*Typha latifolia*	5		4	7	5	6	6
*Veronica beccabunga*	1				5		
Number of species		2	12	11	8	7	7
MIR		18.0	39.3	39.5	36.6	27.6	27.9
Ecological status		poor	good	good	good	moderate	moderate

Chemical parameters of surface water quality differed statistically between test points, which resulted from the type of water flow (Table 3). The reaction of water in the tested watercourses was neutral or slightly alkaline (pH 6.9–7.7). The electrolytic conductivity of rivers did not exceed 570 μS·cm^−1^, which is the limit value for the quality class II. In the case of ditches and the canal, it reached the values ​​of 704 and 809 μS·cm^−1^, respectively. There is a statistically significant relationship between EC and TSS (Grzywna & Sender, 2021). Therefore, very high TSS concentrations were observed in the ditches and the canal (higher than 40 mg·l^−1^). In the canal and rivers, the COD value was below 30 mg·l^−1^. In the ditches, the value of this parameter exceeded 60 mg·l^−1^, which is characteristic of anaerobic conditions. The high COD value was closely related to the low DO values, which in the ditches were lower than the value of 7.5 mg·l^−1^ (the limit for quality class II). A similar situation was observed in the case of nutrients. In the rivers and the canal, the N-NH_4_ content was lower than 0.42 mg·l^−1^, which allowed the water to be classified as quality class II. In turn, in the ditches it sometimes exceeded the value of 0.6 mg·l^−1^, which is characteristic of eutrophic waters. Low values ​​of biogenic indicators in rivers resulted from extensive agricultural activity (semi-natural monotone meadows). The increase in their content resulted from the processes of plant decomposition and peat mineralization in non-flow ditches.

### Relationships between macrophyte species and abiotic characteristics

The relationships between macrophyte species and physical and chemical water parameters were widely disparate (Table 4). Overall, the most significant correlations were observed for EC, TSS and P-PO_4_, which suggests that these variables may play a crucial role as determinants of the presence of macrophyte species in the anthropogenic watercourses. Helophytes, especially the species *Acorus calamus*, *Calla palustris*, *Carex rostrata*, *Equisertum palustre* and *Glyceria maxima*, showed significant correlations with most of the environmental variables. A substantial influence of environmental parameters was also observed for two pleustophytes, *Hydrocharis morsus-ranae* and *Lemna minor*.

**Table 3:** Physical and chemical parameters of water

	DC	DD_1	DD_2	DD_3	RR_1	RR_2	Class II
T (^o^C)	18.4	21.4	19.4	21.3	22.4	21.1	-
pH	7.7	7.3	6.9	7.3	7.3	7.6	-
TSS (mg·l^−1^)	54.5	44.8	42.1	48.0	7.4	7.6	-
EC (µS·cm^−1^)	809	704	692	690	249	314	< 570
COD (mg·l^−1^)	14.9	68.3	80.5	64.3	12.2	26.5	-
DO (mg·l^−1^)	5.1	4.5	3.8	3.1	6.4	5.8	> 7.5
TN (mg·l^−1^)	1.5	2.3	2.4	2.5	1.5	1.9	< 3.5
N-NH_4_ (mg·l^−1^)	0.24	0.61	0.82	0.92	0.31	0.46	< 0.42
N-NO_3_ (mg l^−1^)	0.56	0.77	0.76	1.01	0.42	0.45	< 2.10
TP (mg·l^−1^)	0.52	0.71	0.61	0.59	0.21	0.29	< 0.33
P-PO_4_ (mg·l^−1^)	0.31	0.51	0.44	0.38	0.13	0.19	< 0.10
Chl (µg·l^−1^)	33.4	36.0	35.6	52.7	7.9	9.6	-

**Table 4:** Relationships between environmental parameters and macrophyte species

	HIR	pH	TSS	Con	DO	TN	N-NH_4_	N-NO_3_	TP	P-PO_4_	Chl	COD
*Acorus calamus*	ns	-0.71**	ns	ns	-0.65**	0.76**	0.78**	ns	ns	-0.66**	ns	ns
*Calla palustis*	ns	-0.71**	ns	ns	-0.67**	-0.61**	0.76**	ns	ns	-0.67**	ns	ns
*Carex rostrata*	ns	-0.64**	-0.65**	-0.54*	-0.46*	ns	ns	ns	0.66**	-0.64**	ns	ns
*Carex vesicaria*	ns	ns	ns	ns	ns	ns	ns	ns	0.63**	ns	-0.50*	ns
*Equisetum palustre*	ns	-0.68**	ns	ns	-0.65**	-0.61**	0.76**	ns	ns	-0.66**	ns	ns
*Glyceria maxima*	ns	-0.64**	-0.65**	-0.55**	-0.56**	ns	ns	ns	0.64**	-0.65**	ns	ns
*Juncus effusus*	ns	ns	ns	ns	ns	ns	ns	ns	0.63**	ns	ns	ns
*Mentha aquatica*	ns	ns	ns	ns	0.53*	ns	-0.29*	0.82**	ns	ns	ns	ns
*Phragmites australis*	ns	ns	ns	-0.46**	ns	0.94**	ns	ns	ns	ns	ns	ns
*Rumex hydrolapathum*	ns	ns	-0.68**	-0.57**	ns	ns	ns	0.54*	ns	ns	ns	ns
*Typha latifolia*	ns	-0.73**	-0.63**	-0.83**	-0.46**	0.69**	ns	ns	ns	-0.55**	ns	-0.81**
*Veronica beccabunga*	ns	ns	ns	ns	0.51*	0.53*	ns	0.81**	ns	ns	ns	ns
*Hydrocharis morsus*	ns	-0.60**	-0.65**	-0.54*	ns	ns	ns	ns	0.68**	-0.61**	ns	ns
*Lemna gibba*	ns	ns	ns	ns	ns	0.52*	ns	-0.57**	ns	ns	ns	ns
*Lemna minor*	0.56**	-0.57**	-0.51*	-0.73**	-0.52*	ns	0.68**	ns	0.51*	-0.51**	ns	0.91**
*Lemna trisulca*	ns	-0.62**	ns	-0.67**	-0.50*	ns	0.81**	ns	ns	-0.51*	ns	0.85**
*Nuphar lutea*	0.74**	ns	ns	ns	ns	ns	ns	ns	ns	ns	ns	0.49*
*Potamogeton natans*	ns	ns	ns	ns	ns	ns	ns	ns	0.63**	ns	-0.50*	ns
*Ceratophyllum demersum*	0.53*	0.69**	0.59**	0.68**	ns	ns	ns	ns	ns	0.63**	ns	ns
*Elodea canadensis*	ns	ns	ns	ns	0.63**	ns	ns	0.88**	ns	0.61**	-0.58**	ns
*Potamogeton pectinatus*	ns	0.59**	0.57**	0.79**	ns	ns	-0.56**	ns	ns	ns	ns	-0.78*
*Ranunculus circinatus*	ns	-0.71**	ns	ns	-0.93**	ns	0.73**	-0.69**	-0.41*	-0.86**	0.76**	ns
*Stratiotes aloides*	ns	ns	ns	ns	ns	ns	ns	ns	0.63**	ns	-0.50**	ns

*-significant at *p* < 0.05; **-significant at *p* < 0.01; ns-not significant

We observed a significant effect of width, canal modification, flow type and bottom substrate on the presence of macrophyte species (tab. 1). These variables define the physical niches in rivers and may exert a negative impact on macrophyte community structure (Baattrup-Pedersen et al., 2006; O`Hare et al., 2010; Zelnik et al., 2020, 2021). In our study, the lowest abundance and species richness of macrophytes were observed in canal. The drainage canal was modified using flagstones, which prevent colonization by emergent macrophytes. We noted a significant effect of channel modification on the presence of two helophytes, *Typha latifolia* and *Phragmites australis*. The highest cover of macrophytes was noted at ditches that were characterized by a muddy bottom, no perceptible water flow and the lack of severe bank modification (Table 1). Moreover, we noted positive relationships between the presence of these species with the nutrient content (Table 4). Both species are common in shallow emergent zones along lakeshores and in ditches. Their rapid growth is usually observed in disturbed, high-nutrient environments (Wetzel & van der Walk, 1998; Obarska-Pempkowiak et al., 2002; Tarkowska-Kukuryk, 2006, 2013; Swanson et al., 2017, Jóżwiakowski et al., 2018; Tarkowska-Kukuryk & Toporowska, 2021). Hydromorphological variables, flow type, channel modification and shading affected the presence of the pleustophytes *Lemna minor* and *Lemna trisulca*. The highest cover of these species was observed in drainage ditches. These ecosystems had high nutrient concentrations conducive to free-floating macrophyte dominance (Netten et al., 2011). Moreover, these pleustonic species are able to create dense mats on the water surface and negatively affect the dissolved oxygen concentration in the water (Ceschin et al., 2020; Mäkelä et al., 2004; Rather & Dar, 2020; Takamura et al., 2003). In the ditches ecosystems, we observed very low oxygen concentrations (< 5 mg l^−1^) at sites densely overgrown with *Lemna* spp. The effect of morphological parameters on the presence of elodeids was species-specific. Significant correlations with channel modification, flow type and shading were also observed for elodeids—*Ceratophyllum demersum*, *Elodea canadensis* and *Potamogeton pectinatus*. Shading by woody vegetation, a high proportion of artificial banks and current velocity have been stressed as the most important variables negatively affecting species richness of macrophytes in streams (Lacoul et al*.*, 2006; Hrivnák et al., 2010, 2012; García-Girón et al., 2020). Moreover, the presence of these species was correlated positively with concentrations of phosphate and total nitrate in the water. As an unrooted macrophyte species, *Ceratophyllum demersum* requires nutrient uptake directly from the water column and may compete successfully with phytoplankton (Amorim & Moura, 2020; Mjelde & Faafeng, 1997). This species is often present in hypertrophic waters, where it forms free-floating mats (Melzer, 1999; Seelen et al., 2021). *Potamogeton pectinatus* can tolerate eutrophic conditions. The plant forms a canopy to exploit light near the water surface, but does not produce high biomass (Søndergaard et al., 2017). *Elodea canadensis* is typical of back-flowing and meso-eutrophic streams with medium-to-high nutrient load and higher water transparency and oxygen content (Schneider & Melzer, 2003; Šraj-Kržič et al., 2007; Zelnik et al., 2020), but the species also belongs to the group of macrophytes tolerant of habitat degradation. *Elodea canadensis* is also tolerant of other types of human impact, such as organic pollution and weed cutting (O’Hare et al., 2010). We also noted that the presence of the elodeid species *Stratiotes aloides* has not been significantly affected by any of the hydrological parameters. *Stratiotes aloides* showed a positive correlation with total phosphorus and a negative relationship with chlorophyll-*a* concentration. The species is regarded as an important indicator of the ecological conservation state of ditch ecosystems (Zantout et al., 2011) and has potential as a conservation surrogate, since plant aggregations maintain diverse macroinvertebrate communities (Ceschin et al., 2020; Tarkowska-Kukuryk, 2006, 2013). *Stratiotes aloides* forms typical associations with *Hydrocharis morsus-ranae* and with plant species such as *Lemna trisulca* and *Potamogeton* spp (Strzałek & Koperski, 2009; Strzałek et al., 2019).

In Poland, in 1989, there was a change in the political system, which contributed to a sharp reduction in cattle breeding in small farms. The decline in livestock production resulted in the abandonment of the use of meadows and the lack of exploitation of water systems (Cegielska et al., 2018; Swinnen & Vranken, 2010). The lack of maintenance of the ditches contributed to the succession of the *Salix viminalis*, *Urtica dioica L.* and *Phragmites australis* as well as silting of the watercourses. These factors significantly contributed to the subsidence of peatlands and the simplification of the species composition of vegetation. In our study, the average number of species was 7 and ranged from 2 to 12. The most common were: *Ceratophyllum demersum, Lemna minor, Phragmites australis,* and *Typha latifolia*. For this reason, contrary to expectations, no rare or endangered plant species were found. The current conditions in the studied sections of the watercourses are not suitable for the succession of endangered plant species. Unfavorable conditions for plant development result from large fluctuations in water levels in watercourses and mineralization of peatlands (Grzywna, 2017; Grzywna & Kowalczyk-Juśko, 2018). Due to the large distances from the place of residence and the use of ready-made fodder in cattle breeding, there are no artificial watercourses subject to intensive conservation in the Western Polesie. In this region, all ditches and canals have been abandoned by people and are undergoing spontaneous succession.

## Conclusions

MIR values showed high variability among the anthropogenic watercourses studied (regulated rivers, drainage ditches, and drainage canal). The results of our study showed that the drainage ditches had favorable habitat conditions (good ecological status) for macrophyte communities in comparison with the regulated rivers and drainage canal. Both the species richness and percentage cover of macrophyte species were highest in the ditches ecosystems. The favorable conditions for macrophytes in the drainage ditches, who demonstrated that ditches can support high diversity of macrophyte species.

Overall, macrophyte species respond very clearly to variations in hydromorphological and environmental conditions (nutrients, oxygen and planktonic chlorophyll-a) with changes in their abundance and richness. The results indicate that macrophyte communities should be recognized as indicators of the ecological status of artificial and modified watercourses (ditches, canals and regulated rivers). The relevance of the methods used in the study should be verified on a larger number of sites.

## Data Availability

All data generated or analyzed during this study are included in this published article (and its supplementary information files).
